# Immune‐mediated drug‐induced liver injury secondary to Omeprazole: A case report

**DOI:** 10.1002/ccr3.3421

**Published:** 2020-10-27

**Authors:** Sara Seife Hassen, Fateen Ata, Ammara Bint I Bilal, Mohamed Salih Ali, Mahir Petkar, Abdelnaser Y. Awad Elzouki, Muhammad Zahid

**Affiliations:** ^1^ Department of Internal Medicine Hamad General Hospital Hamad Medical Corporation Doha Qatar; ^2^ Department of Radiology Hamad General Hospital Hamad Medical Corporation Doha Qatar; ^3^ Department of Pathology Hamad General Hospital Hamad Medical Corporation Doha Qatar

**Keywords:** autoimmune hepatitis, drug‐induced liver injury, omeprazole, proton pump inhibitors

## Abstract

Omeprazole is a rare cause of DILI with autoimmune hepatitis features and should be considered when seeing patients with acute liver injury. The causative drug should be promptly identified and discontinued to avoid any permanent liver damage.

## Graphical abstract

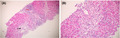

## 1 INTRODUCTION

The autoimmune pattern of liver injury is infrequent with PPI and has not been reported before with omeprazole. We describe a case of omeprazole induced liver injury in a 72‐year‐old female with no known previous hepatic disease. Discontinuation of the drug resulted in the resolution of liver injury clinically and biochemically.

Drug‐Induced liver injury is one of the most common causes of acute liver injury, accounting for nearly 10% of acute hepatitis cases in the United States.^1^ DILI’s annual incidence ranges from 10 to 15 per 10 000 to 100 000 persons exposed to prescription medications.^2^ DILI is more common in females and those with malnutrition and a history of alcohol abuse. The majority of patients recover once the offending medication is stopped. Patients with bilirubin more than twice the normal limit and Alanine aminotransferase (ALT) more than three times the upper limit secondary to DILI carry a poor prognosis.^3^ Autoimmune hepatitis is another well‐known cause of liver injury, with literature dating back to 1951.^4^ Sometimes, patients can present with liver damage secondary to drug‐induced liver injury with an autoimmune pattern. The autoimmune pattern of liver injury secondary to drugs has been further subclassified in the literature depending on the pattern of injury, presence, or absence of auto antibodies and histologic features, various subclasses of autoimmune pattern of DILI.^5^

Hepatotoxicity due to omeprazole is a rare entity.^6^ Various other medications have been described as common causes. DILI can have dire consequences, with rare progression to acute liver failure requiring transplantation.^7^ Although there are studies into serologic and molecular biomarkers DILI,^8^ and several scoring systems,^9^ clinical practice is guided mostly by expert opinion.

## 2 CASE REPORT

A seventy‐two‐year‐old female, a known case of Type 2 Diabetes, hypertension, and subclinical hyperthyroidism, presented with jaundice associated with dark urine, pale stool, fatigue, and poor appetite for 2 weeks. She did not complain of fever, abdominal pain, or vomiting. She also denied itchiness, hematemesis, melena, or significant weight loss. Her surgical history was unremarkable. She was compliant with her diabetic medications, ie, metformin and Sitagliptin. Two weeks before the presentation, the patient was seen in primary healthcare with mild dyspepsia for 1 month. She tested negative for Helicobacter Pylori was prescribed omeprazole 20 mg daily for 4 weeks, to which she was compliant. She had no prior gastric complaints and had not taken proton pump inhibitors before.

The patient did not have a history of smoking or alcohol intake and had no known allergies. She was not taking any herbal or over the counter medicines. She denied a family history of liver or biliary disease and malignancies. None of her other family members had similar symptoms. She did not have any recent travel outside Qatar.

On physical exam, she was afebrile (36.7°C), with a blood pressure of 142/79 mm Hg, pulse rate 88 beats/minute, respiratory rate 16 breaths/minute, and oxygen saturation 97% on room air. She had yellow discoloration (jaundice) of her eyes. There was no lymphadenopathy or organomegaly, and the rest of the system examinations were unremarkable.

Basic labs revealed a raised total and direct bilirubin, raised ALP, ALT, and AST (Table 1). Complete blood profile, renal function, serum electrolytes, including sodium, potassium, calcium, magnesium, and INR, were normal (Table 1). Ultrasound of the abdomen showed a 13 cm liver with normal echotexture. No lesions or Intra Hepatic Biliary Radicle dilatation were noted. The common bile duct was 2.6 mm. Hepatitis A and E IgM antibodies, hepatitis B surface antigen, and anti‐hepatitis C antibody, anti – HIV antibodies were negative. Additionally, CMV and EBV PCR were negative. The patient's biochemistry was favoring the possibility of AIH vs DILI. The patient's medications were carefully reviewed. As omeprazole was the only medicine recently started, and with some scarce evidence in the literature, it was suspected to be the culprit drug and was thus discontinued on day 3.

**Table 1:** Investigations of the patient including blood counts and metabolic panel on admission, discharge and follow‐up

Investigation	Admission	Discharge	Follow‐up	Normal range
White cell count	8.3	6.7	‐	4‐11 × 10^9^/L
Hemoglobin	12.6	11	‐	12‐15 g/dL
Platelets count	215	249	‐	140‐450 × 10^5^/L
INR	1.1	1	‐	‐
Urea	2.3	3	‐	2.1‐8 mmol/L
Creatinine	82	34	‐	44‐100 μmol/L
Sodium	134	137	‐	135‐145 mmol/L
Potassium	3.9	4.2	‐	3.5‐5.2 × 10^9^/L
Bicarbonate	22	24	‐	22‐29 mg/dL
Ph	7.41	‐	‐	7.35‐7.45
Bilirubin total	212	90	12.8	0‐21 mg/L
Bilirubin Direct	188	88	‐	<5.1 μmol/L
Alkaline phosphatase	192	140	119	35‐104 U/L
GGT	376	‐	‐	5‐42 U/L
ALT	342	174	10.5	0‐41 U/L
AST	444	180	18	0‐40 U/L
R Factor	4.5	‐	‐	‐

As the patient's liver enzymes showed a mixed picture of cholestasis and hepatitis without any obvious obstruction of the biliary tract, an MRCP was arranged. Pancreas and biliary tree were unremarkable on MRCP, with no evidence of any underlying neoplastic process. However, there was periportal edema and trace of perihepatic free fluid, suggestive of diffuse liver disease.

On day four, the AIH work up was back. ANA and ASMA were positive with titers of 1:160 (nucleolar pattern) and 1:80, respectively. IgG (22, NR 7‐16 gm/L) and IgG subclass 1(1370, NR 405‐1011 mg/dL) were raised. The rest of the AIH workup, including AMA, ALKM, ANCA, APLAR‐2 AB, anti – MPO, and anti‐PR – 3, was unremarkable.

**FIGURE 1:**
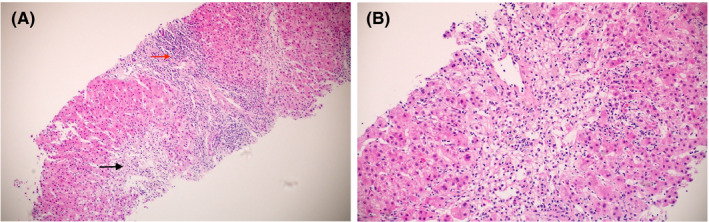
Histopathology of Liver biopsy: (A). Liver biopsy showing perivenular zone 3 necrosis (black arrow) and moderate portal tract inflammation (red arrow) (H and E ×4), (B). High power view displaying marked centrilobular necrosis (H and E ×20)

Although the liver enzymes were slowly declining (Table 1) but were still considerably high; hence on day 10, an ultrasound‐guided liver biopsy was done. The histopathology (Figure 1) revealed confluent zone 3 necrosis with moderate portal tract inflammation and focal lobular inflammation: features highly suggestive of DILI, and not entirely compatible with autoimmune hepatitis.

The patient's liver enzymes kept trending down during her hospital stay. Her condition did not require steroid treatment. Gradually her jaundice improved, and the patient was discharged with a follow‐up in medicine and gastroenterology clinic.

The patient was seen in an acute medicine clinic for follow‐up after 1 month. She had no residual jaundice, and liver enzymes had returned to normal (Table 1). A repeated ANA, ASMA, and ALKM titer were negative, suggesting the diagnosis as AIH‐DILI secondary to omeprazole.

## 3 DISCUSSION

**FIGURE 2:**
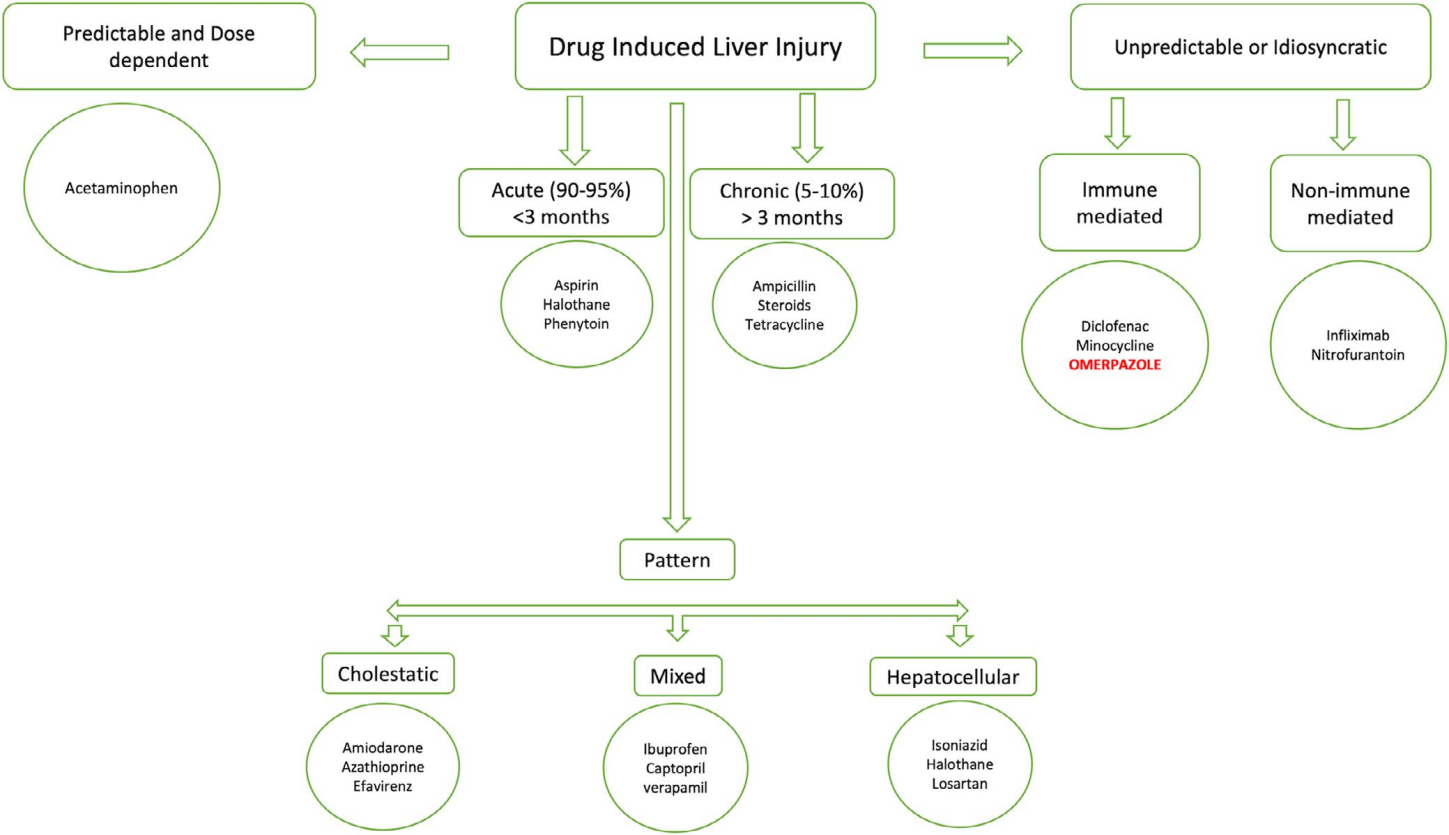
Classifications of DILI

DILI is considered one of the most frequent causes of acute liver injury in the developed world and is also the most frequent reason behind the withdrawal of drugs from the market.^10^, ^11^ One of the reasons for it being a common presentation is the difficulty to test this effect in phase III trials. Most of the drug‐induced liver injuries are detected in postmarketing studies, ie, phase IV clinical studies. DILI can be divided into predictable or unpredictable, acute, or chronic, based on the pattern of liver injury (hepatic, cholestatic, or mixed), as shown in the flowchart (Figure 2) with some examples.^12^

Clinical presentation of DILI varies with the causative drugs. Generally, the most common presenting feature is jaundice, as in our case. Chronic liver disease features are hardly seen in initial presentations. Two main clinical patterns of liver injury that can be differentiated effectively on biochemistry are hepatitis and cholestasis. Aminotransferases are higher than alkaline phosphatase in the hepatitis pattern for liver disease, while a reverse pattern is seen in the cholestatic picture. A more precise judgment can be made via *R*‐value calculation (“[patient's ALT/upper limit of normal ALT]/[patient's ALP/upper limit of normal ALP]”), a value of or above 5.0 indicates a hepatocellular injury, *R* < 2.0 indicates a cholestatic injury, a value 2.0 to 5.0 indicates a mixed pattern.^13^ Our patient had an R‐value of 4.5, revealing a mixed pattern of liver injury.

Various risk factors have been linked to DILI in previous studies, including female sex, old age, dosage, and duration of exposure with the causative drug. Whether a previously present liver injury is a superadded risk for the development of DILI is debatable.^14^

One of the comparatively less studied DILI classification is the immune‐mediated and nonimmune‐mediated injury. In the immune‐mediated pattern, the drug or its components trigger the adaptive immunity to result in organ damage. On the contrary, in the nonimmune‐mediated pathway, damage‐associated molecular pathway (DAMP) proteins result in direct organ injury.^15^ Immune‐mediated DILI usually occurs within 1‐6 weeks of the exposure, whereas nonimmune‐mediated DILI can occur from 1 month to 1 year of exposure.^14^

Immune‐mediated DILI can be further subdivided based on the mechanisms of immune mediation. They are:

1. Autoimmune hepatitis (AIH) with DILI (drug‐induced injury in known autoimmune hepatitis).
2. DILI‐AIH (patients with no previous diagnosis of AIH in whom the drug causes a chronic autoimmune process).
3. Immune‐mediated (IM)‐DILI (this is due to autoimmune hypersensitivity reaction with negative AIH ABs).
4. A mixed autoimmune type (patients with combined characteristics of the DI‐AIH and IM‐DILI).
5. DILI with positive autoantibodies.^5^

Clinical diagnosis of Autoimmune liver injury can be made if the patient has; liver enzymes more than twice the UNL with raised Immunoglobulin – G or one positive AB (including ANA/ASMA (≥1:40 titer), Anti‐LKMA, anti‐Liver Cytosol antibody 1, or antisoluble liver/liver pancreas antibodies) and exclusion of Alcoholic, viral, or other infectious causes of liver injuries.^16^ Furthermore, AIH scoring systems have been validated in previous studies. Hennes et al introduced one of the simplified AIH scoring systems, shown in Table 2.^17^ Our patient had a score of 7, thus favoring the diagnosis of AIH. However, the histopathology findings in our patient's biopsy were not typical of AIH and were more in favor of DILI. Additionally, many important antibodies seen in AIH were negative in our patients such as Anti‐LKMA and AMA. ANA and ASMA were positive, but titers were not very high. These collective findings were suggestive of a mixed pattern of liver injury.

**Table 2:** Simplified diagnostic criteria for autoimmune hepatitis, Adopted from Hennes et al (<6 = AIH unlikely, 6 = Probably AIH, ≥7 = definite AIH)

Variable	Cutoff	Score
ANA or SMA	≥1:40	+1
ANA or SMA	≥1:80	+2
or LKM	≥1:40	+2
or SLA	Positive (any titer)	+2
IgG	>Upper normal limit	+1
IgG	>1.10 times upper	+2
Liver Histopathology (evidence of hepatitis)	Compatible with AIH	+1
	Typical AIH	+1
	Atypical AIH	+2
Absence of viral hepatitis	Yes	+2

Diagnostic challenges arise when detailed workup is unrevealing of the cause of liver injury. A drug‐induced liver injury should always be in the differential diagnosis of acute liver injury, even if the offending medicine is not commonly known to cause DILI. Using this approach, omeprazole was suspected to be the culprit, despite being a rare cause of DILI. This was supported by the histopathologic features going more with drug‐induced liver injury, and more significantly, a marked resolution of liver injury upon discontinuation. Therefore, the patient falls into the last category, ie, DILI, with positive antibodies. Some of the drugs known to cause autoimmune type DILI are clometacin, diclofenac, fenofibrate, methyldopa, minocycline, nitrofurantoin, papaverine, phenytoin, propylthiouracil, and statins, among others.^12^ PPI are also associated with DILI, with a few reported cases.^18^, ^19^, ^20^, ^21^, ^22^, ^23^, ^24^, ^25^, ^26^, ^27^, ^28^, ^29^, ^30^, ^31^, ^32^ Although omeprazole is the most common PPI associated with DILI, to the best of our knowledge, it has not been reported with the AIH DILI pattern. Previously reported patterns of DILI with omeprazole are pure hepatocellular, cholestatic, and mixed hepatitis with negative antibodies.^21^, ^24^, ^25^, ^26^, ^27^, ^28^, ^29^

Establishing a diagnosis begins with clinical history and examination, supported by laboratory and imaging and tissue biopsy. Although tissue biopsy is not necessarily required in the workup of DILI, combined with the clinical suspicion, it aided in establishing a diagnosis in our patient.^33^ Awareness of common and rare drugs known to cause liver injury is imperative to the management of DILI, guiding the offending agent's timely withdrawal. Various medications have been used in acute liver injury. Although classically NAC is used as an antidote, and primarily for acetaminophen overdose, the literature describes others such as activated charcoal for decontamination, Ursodeoxycholic acid for reducing time to recovery, and cholestyramine for symptomatic management.^34^ Acute liver failure is a rare and potentially fatal complication of liver injury in the absence of liver transplantation.^7^ Cautious use of hepatotoxic drugs is the best way to avoid the development of DILI, while early recognition and withdrawal of the offending agent being paramount to prevent further injury and liver failure.

Although liver biopsies can play a vital role in the diagnosis, injuries attributed to drugs’ effects can be notoriously tricky to interpret, especially when DILI is not suspected clinically. DILI can show a wide variety of histologic features, classified into either necro inflammatory or cholestatic injury.^5^ These patterns of injury are also seen in a variety of other clinical conditions, including acute and chronic viral hepatitis, autoimmune hepatitis, primary biliary cholangitis, and significant duct obstruction, among others, contributing to the interpretative dilemma.^33^ The reporting pathologist must suspect DILI when the histologic changes are not entirely compatible with the patient's history.

In our patient, the biopsy revealed confluent zone 3 necrosis with moderate portal tract inflammation and focal lobular inflammation. With positive autoantibodies, it was reasonable to suspect autoimmune hepatitis clinically. Although described in the centrilobular variant, zone 3 necrosis is an uncommon histologic feature of autoimmune hepatitis. In such a scenario, despite positive autoantibodies, DILI was more likely.

Although ANA and ASMA were positive in our case, and the initial impression was of AIH, the histopathologic picture was suggestive more of a drug‐induced injury. A careful literature review was done to identify potential DILI, as the patient was not on any drug known to have a strong association with liver damage. Omeprazole was discontinued, although there is limited evidence for this medicine to cause DILI. Stopping the possible offending drug resulted in a rapid resolution of liver injury without steroid therapy. Thus, a careful judgment of reversible causes of liver injury proved exceptionally beneficial to the patient.

One of the limitations of this report is that rechallenge with omeprazole was not done, which could have helped to confirm the diagnosis. However, the causality score via Roussel Uclaf Causality Assessment Method (RUCAM) in our patient was 7, which indicates omeprazole was the probable cause, especially in the absence of any other suspicious prescription, over the counter or herbal medicines.

There is a recent interest in investigating novel biomarkers to establish an early causality in DILI. Markers such as Micro RNA 122, Cytokeratin 18 (a cytoskeleton protein), Glutamate Dehydrogenase, Macrophage Colony‐Stimulating Factor Receptor 1, and bile acids are currently being studied. Their sensitivities for detection, monitoring progression, and resolution of DILI have a promising potential.^35^, ^36^ Additionally, studies on major histocompatibility complex have shown the association of specific human leukocyte antigens (HLA) with DILI. HLAs have a high negative predictive value for DILI, indicating a valuable diagnostic role in this regard. All these aspects need further studies and will help minimize the liver damage caused by drugs via an early establishment of association.^37^

## 4 CONCLUSIONS

PPI is one of the rarest causes of DILI with features of autoimmune hepatitis. DILI should be kept in differential diagnosis while seeing patients with acute liver injury. The significance of DILI with an autoimmune pattern is relatively understudied and needs further clinical studies to understand the phenomenon better. The causative drug in DILI should be promptly identified and discontinued to avoid any permanent liver damage.

## CONFLICT OF INTEREST

This manuscript is original work and has not been submitted or is not under consideration for publication elsewhere. All the authors have reviewed the manuscript and approved it before submission. None of the authors have any conflict of interest from publishing this work.

## AUTHOR CONTRIBUTIONS

SH: Manuscript writing, literature review, review, and approval of the final manuscript. FA: Manuscript writing, literature review, revisions in manuscript, review, and approval of the final manuscript. AB: Manuscript writing, literature review, review, and approval of the final manuscript. MP: Write‐up of the pathology part of the manuscript, and approval of the final manuscript. MA: Case write‐up, literature review, and approval of the final manuscript. AE: Manuscript revisions, literature review, critical review, and approval of the final manuscript. MZ: Case identification, Manuscript revisions, literature review, critical review, and approval of the final manuscript.

## ETHICAL APPROVAL

This work was approved by Medical Research Center (MRC) Qatar before submission.

## CONSENT

Written informed consent was taken from the patient before the submission of the manuscript.

## Data Availability

Data sharing not applicable to this article as no datasets were generated or analysed during the current study.
